# Unpacking the Trade-Offs: A Meta-Analysis of Soil Fertility, Crop Yield, and Greenhouse Gas Emissions Across Fertilizer Types (Organic, Mineral) and Cropping Systems

**DOI:** 10.3390/plants14193005

**Published:** 2025-09-28

**Authors:** Elnaz Amirahmadi, Mohammad Ghorbani

**Affiliations:** School for Environment and Sustainability, University of Michigan, Ann Arbor, MI 48109, USA; elnaza@umich.edu

**Keywords:** organic amendment, fertilizer management, GHG mitigation, nutrient efficiency, crop yield

## Abstract

Different strategies are used in organic and conventional cultivation, which can significantly influence crop yield, greenhouse gas (GHG) emissions, and soil quality. However, the relative efficiency of these fertilization practices has not been systematically compared. The objective of this study was to evaluate the impacts of organic, conventional, and semi-organic fertilization systems on soil properties, crop productivity, and GHG emissions through a comprehensive meta-analysis. The analysis showed that conventional systems had the highest increase in nitrous oxide (N_2_O) emissions (+62%), followed by semi-organic (+55%) and organic (+21%). Soil texture strongly influenced methane (CH_4_) and carbon dioxide (CO_2_) fluxes, with clay soils showing the highest CH_4_ response (+50%). Cropping practices such as intercropping and crop rotation enhanced soil nitrate availability (+18%), while vegetable and cereal systems improved crop yield by +29% and +19%, respectively. Importantly, semi-organic systems increased yield (+25%) while reducing greenhouse gas intensity (+13%), especially in cereals under intercropping. Integrating organic inputs into semi-organic systems, especially in cereal cultivation under intercropping practices, appears to reduce the carbon intensity per unit yield while maintaining productivity. These findings underscore the importance of context-specific management strategies to optimize agronomic performance and mitigate environmental impacts.

## 1. Introduction

Advancements in agricultural technologies have led to increased productivity, but they have also contributed substantially to greenhouse gas (GHG) emissions. Data from the Food and Agriculture Organization (FAO, 2022) indicate that emissions linked to crop production and food systems have risen by approximately 17% over the past three decades. Additionally, soil characteristics shaped by varying agricultural practices play a crucial role in regulating GHG emissions through their influence on microbial activity [1]. Among the soil parameters, soil organic carbon and total nitrogen are crucial soil fertility parameters that determine the intensity and abundance of soil microbial communities, and plant growth [2]. Various cropping methods—such as monocropping, intercropping, and crop rotation—can influence nutrient dynamics and greenhouse gas emissions, particularly when paired with different fertilization regimes including fully synthetic (conventional), organic, or integrated approaches that combine both. While chemical fertilizers and agrochemicals are known to boost plant nutrition and yield, their excessive application, especially under continuous monoculture systems, can lead to a decline in soil fertility [3]. Moreover, intensive tillage—common in conventional farming—can accelerate soil degradation, reduce nutrient retention, and elevate GHG emissions [4]. It is also important to consider that conventional agricultural inputs contribute to indirect emissions through the energy-intensive production of fertilizers, fossil fuel use, and reliance on machinery [5].

It might not help to generalize terms such as “organic” and “conventional” to better comprehend the soil–plant interactions, since there are several management procedures that are different between organic and conventional soil nutrient management including the type of fertilizer used and the methods used to control soil and plant stresses. However, organic fertilization refers to using organic sources of essential nutrients for plant growth, whereas the conventional approach primarily uses inorganic nutrient supplies. In organic farming systems, the use of chemical agents for weed and disease control is restricted, unlike in conventional practices, where such inputs are commonly applied [6]. In contrast to conventional fertilization, which potentially could assist in environmental adverse, organic amendments are environmentally friendly regarding emissions, soil erosion, biodiversity pressure, and energy consumption, with positive effects on soil and water quality [7,8]. It has been reported that the constant addition of compost, manure, and crop residues in an organic farming system provides a greater soil organic matter (SOM) [9] and fewer GHG emissions [10,11,12]. One major limitation of organic agriculture is its relatively low land use efficiency. Studies have shown that organic systems may require up to 84% more land compared with conventional farming methods [13], primarily due to yield reductions that typically range from 20% to 34% below those of conventional systems [14]. In particular, reduced yields in organic systems often stem from slower nutrient mineralization from organic inputs, limited availability of readily accessible nitrogen, and restrictions on synthetic crop protection products. While organic farming offers clear environmental benefits, such yield penalties raise concerns about its scalability in the context of global food security, especially under increasing demand for staple crops [15]. Expanding organic production without addressing these efficiency gaps could therefore result in greater land conversion pressures, potentially undermining biodiversity conservation and climate mitigation goals [16]. Regarding finding an optimal rate of nutrient inputs without high nutrient losses and emissions as well as maximizing the yield per unit area, semi-organic agriculture is considered a mediocre approach [17]. Semi-organic agriculture is a combination of conventional and organic fertilization for effective nutrient management as one of the fundamental requirements of sustainable agriculture and could offer a potential solution for long-term nutrient management with lower GHG emissions [18]. In practice, semi-organic systems allow for the strategic use of synthetic fertilizers to supply immediate plant nutrient demands, particularly nitrogen, while simultaneously incorporating organic amendments that improve soil organic matter, microbial activity, and long-term fertility. This dual input strategy has been shown to stabilize yields across different soil types and cropping systems, reduce nutrient losses through leaching or volatilization, and lower the greenhouse gas intensity per unit yield. Unlike fully conventional farming, which often prioritizes short-term yield at the cost of soil health, or fully organic farming, which can compromise yield stability, semi-organic approaches balance these trade-offs [19,20].

To gain a deeper comprehension of the dynamics of nutrients between soil, plants, and atmosphere, the primary focus should be on the way various soil characteristics and management approaches affect certain biological, chemical, and physical alterations in agricultural fields. Given the variability in findings across the literature, a thorough evaluation of conventional, organic, and integrated fertilization practices is essential. To date, no single study has simultaneously assessed multiple agricultural systems with varying cropping strategies concerning their impacts on GHG emissions, soil characteristics, and crop performance. This gap strongly emphasizes the need for a meta-analysis that can integrate and compare results across diverse fertilization and cropping systems, accounting for variations in soil types, plant species, and management practices, to provide a clearer and more comprehensive understanding of their relative impacts on greenhouse gas emissions, soil health, and crop productivity. Such a synthesis is essential to guide evidence-based recommendations for sustainable agricultural management and identify strategies that optimize both productivity and environmental outcomes. Therefore, this study conducted a meta-analysis to offer a clearer understanding of how farming practices, cropping approaches, and inherent soil conditions affect greenhouse gas emissions, carbon indicators, soil health, and crop productivity. To do this, the effects of (i) three fertilization strategies (conventional, organic, semi-organic), (ii) three soil textures (sandy, loamy, clay), (iii) three cropping strategies (sole cropping, intercropping, crop rotation), (iv), and three types of plants (cereal, vegetable, orchard) on GHG emissions, carbon indices, soil properties, and crop yield were meta-analyzed comprehensively.

## 2. Results

### 2.1. Changes in GHG Emissions

**Figure 1:**
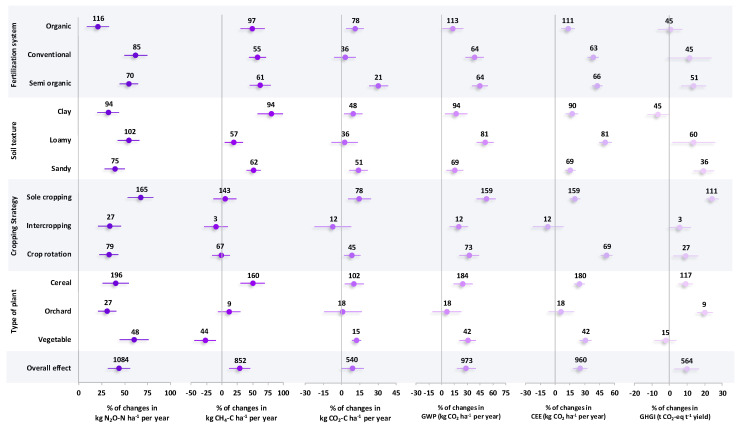
Percentage changes of GHG parameters affected by a variety of effective factors including fertilization systems, soil textures, cropping strategies, and types of plants. Subgroups with overlapped CIs on the vertical zero line suggest no significant change in property. Subgroups with overlapping CIs with each other are not significantly different.

The results of the meta-analysis showed that the GHG parameters were affected significantly (*p* < 0.05) by different fertilization systems and soil texture (Figure 1). In detail, organic fertilization resulted in N_2_O emission with a positive effect size of 20.7%. However, this amount was far less than conventional and semi-organic fertilization systems, which caused a positive effect size of 62.1% and 54.7%, respectively. Also, all three types of fertilization systems resulted in a 56.6% increase in CH_4_ on average. Among the different fertilization systems, semi-organic resulted in the highest CO_2_ emission with a positive effect size of 30.5%. Without significant difference with each other, both conventional and semi-organic showed the highest GWP, CEE, and GHGI with 41.7%, 40.7%, and 12.6% effect sizes on average, respectively.

In terms of soil texture, the clay texture demonstrated the highest CH_4_ with a positive effect size of 49.6%. However, other GHG indices were considerably affected by the coarse textures. Loamy texture resulted in the highest N_2_O, GWP, and CEE with positive effect sizes of 54.3%, 50.9%, and 50.8%, respectively. Also, the sandy texture showed a significant effect on increasing CO_2_ and GHGI with a 13.8% and 19.5% effect size, respectively. Among the different cropping strategies, sole cropping resulted in the highest GHG indices with positive effect sizes as follows: N_2_O (67.2%), CO_2_ (14.6%), GWP (52.2%), and GHGI (24.4%). However, intercropping and crop rotation did not show a significant difference from each other in most cases. However, it should be noted that the highest CEE was observed in crop rotation, with a 52.2% positive effect size. In the case of plant effectiveness, the results of the meta-analysis revealed that vegetables and cereals had a considerable effect on increasing N_2_O and CH_4_ in order, with positive effect sizes of 60.3% and 50.1%, respectively. Also, the highest GHGI was obtained from orchard fields with a positive effect size of 20.1%. However, cereal production resulted in a 9.3% effect size in GHGI, which had a significant difference with orchard production.

### 2.2. Changes in Carbon Indices

**Figure 2:**
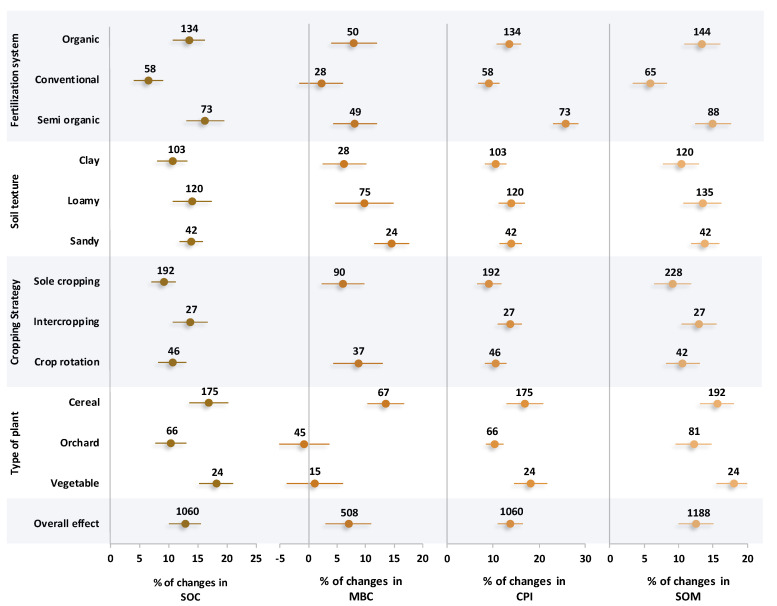
Percentage changes of the soil carbon indices affected by a variety of effective factors including fertilization systems, soil textures, cropping strategies, and types of plants. Subgroups with overlapped CIs on the vertical zero line suggest no significant change in property. Subgroups with overlapping CIs with each other are not significantly different.

Based on the results, different fertilization systems, soil textures, cropping strategies, and plants significantly (*p* < 0.05) changed the status of carbon in soil (Figure 2). In detail, semi-organic and organic systems of fertilization did not show notable differences with each other in most of the C indices, and considerably increased the SOC, MBC, and SOM with 14.9%, 8.1%, and 14.3% positive effect sizes on average, respectively. However, in the case of CPI, semi-organic, organic, and conventional resulted in positive effect sizes of 25.8%, 12.4%, and 9.1%, respectively. Although there was no significant difference among the three types of soil textures in terms of the soil carbon status, an increase in coarse mineral particles of soil resulted in an ascending trend in the soil C indices. Based on the results, clay, loamy, and sandy textures caused an increase in MBC with positive effect sizes of 6.3%, 9.8%, and 14.5%, respectively. On average, loamy and sandy soils resulted in 13.9%, 14.1%, and 13.6% positive effect sizes in SOC, CPI, and OM, respectively. Although there was no significant difference among different crop strategies, the intercropping strategy showed the best performance with the highest SOC (13.7%), CPI (13.6%), and OM (12.9%). In terms of plant type, the highest MBC was observed from cereal cultivation, with a 13.5% positive effect size higher than vegetable and orchard. However, there were no significant differences in terms of SOC between vegetable and cereal, with a positive effect size of 17.6% on average.

### 2.3. Changes in Soil Properties

**Figure 3:**
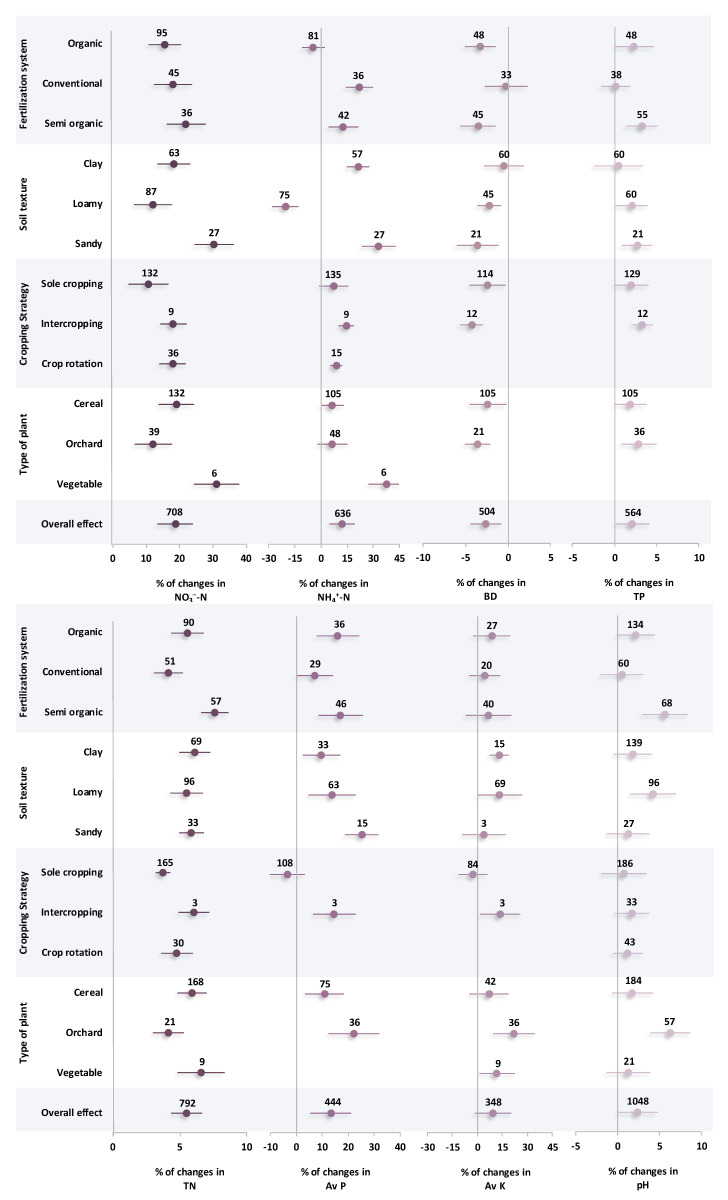
Percentage changes of soil properties affected by a variety of effective factors including fertilization systems, soil textures, cropping strategies, and types of plants. Subgroups with overlapped CIs on the vertical zero line suggest no significant change in property. Subgroups with overlapping CIs with each other were not significantly different.

The results showed that the soil nutrient status was significantly (*p* < 0.05) affected by changes in fertilization systems and cropping strategies (Figure 3). The lowest available nitrate was observed in the organic fertilization system with a 15.5% positive effect size. Even in terms of aluminum availability, conducting organic fertilization resulted in a negative effect size of 4.6%. However, both conventional and semi-organic caused a positive increase in soil ammonium (with positive effect sizes of 21.8% and 12.7%, respectively) without a significant difference with each other. Also, integrating organic amendment in the system of fertilization resulted in an increase in soil porosity with a positive effect size of 2.4% and 3.5% for organic and semi-organic systems, respectively. However, those increases did not statistically differ from the conventional system. In terms of macro nutrient availability, semi-organic caused a positive increase in TN (7.6%), which was significantly higher than the conventional and organic systems of fertilization. In the case of available P and K, there were no significant differences among the three fertilization systems. Also, it should be noted that semi-organic resulted in the highest pH with a positive effect size of 5.7%.

In general, sandy-textured soil showed the highest availability of nitrate and ammonium, with a 30.4% and 33.1% positive effect size, respectively, significantly higher than clay and loamy textures. In terms of macro nutrient availability, although all soil textures showed a positive effect size, there were no significant differences between them. Also, the coarser textures showed an ascending trend in soil porosity and a descending trend in bulk density. However, there was no statistical difference among the three textures. The amount of soil available nitrate considerably increased under intercropping and crop rotation strategies, with positive effect sizes of 18.1% and 17.8%, respectively. The same results were obtained in terms of soil available nitrogen with positive effect sizes of 6.1% and 4.8% for intercropping and crop rotation, respectively. However, there was no significant difference among the three cropping strategies in terms of soil bulk density, porosity, and pH. Based on the results, all three types of plants showed a positive effect size in nutrient availability. However, vegetable cultivation showed a significantly higher nitrate and ammonium availability in soil with positive effect sizes of 31.1% and 37.5%, respectively, in comparison with cereal and orchard. The same trend was observed in terms of TN and Av.K, but no statistically significant difference was observed between vegetables and the two other types of plantations.

### 2.4. Changes in Crop Yield

**Figure 4:**
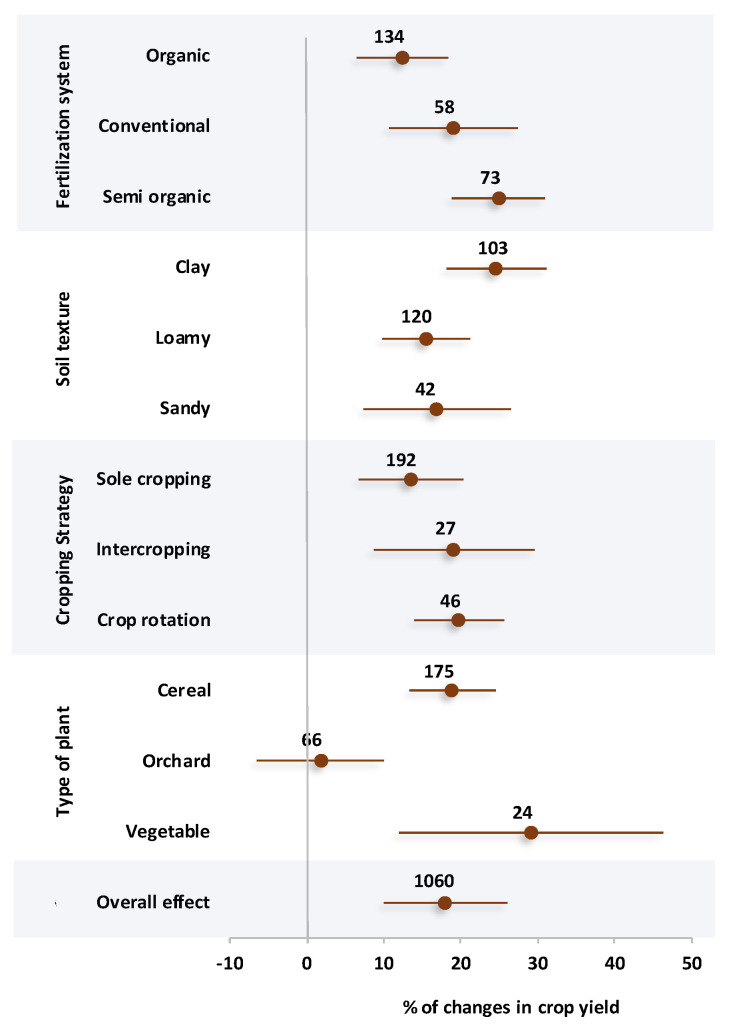
Percentage changes of crop yield affected by a variety of effective factors including fertilization systems, soil textures, cropping strategies, and types of plants. Subgroups with overlapped CIs on the vertical zero line suggest no significant change in property. Subgroups with overlapping CIs with each other were not significantly different.

The percentage changes of crop yield affected by different variables are presented in Figure 4. Based on the results, all systems of fertilization increased crop yield, and the highest effect size was observed in the semi-organic system with a positive percentage change of 24.9%. The second highest crop yield was related to the conventional system of fertilization with a 19.1% positive effect size. However, it should be noted that the semi-organic and conventional systems did not have a statistically significant difference from each other. The organic system of fertilization resulted in a 12.4% positive effect size in crop yield, which had a significant difference from the semi semi-organic system. Among the soil textures, the clay texture soils caused a positive effect size of 24.6% in terms of crop yield. On the other hand, loamy and sandy soils did not show a significant difference from each other, with an average positive effect size of 16.2%. In the case of cropping strategies, all three strategies showed a positive increase in crop yield without a significant difference from each other. However, crop rotation and intercropping showed a better performance with a positive effect size of 19.5% on average. The results of the meta-analysis showed a significantly higher percentage change of crop yield in vegetables and cereals compared with orchards. In detail, vegetable and cereal cultivations resulted in 29.1% and 18.9% positive effect sizes in crop yield, respectively.

### 2.5. Pearson Correlation Between Affected Factors

**Figure 5:**
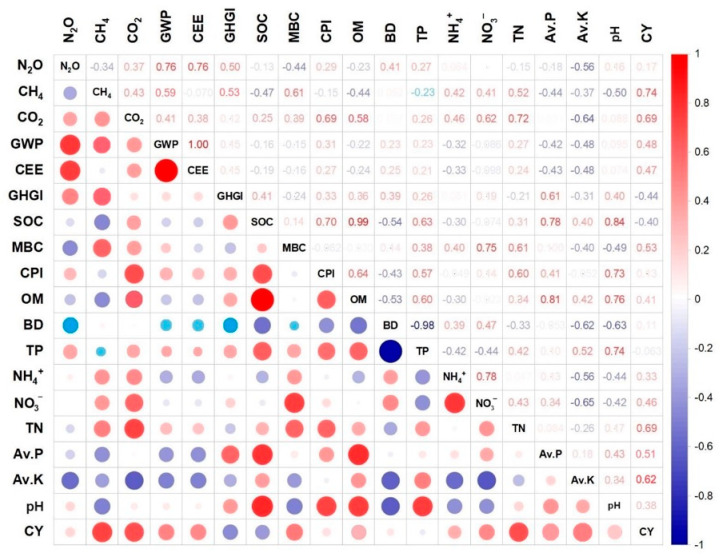
Pearson correlation matrix between different affected properties. Color spectra represent the coefficient of determination (blue and red indicate the intensity of negative and positive correlation, respectively). The significance of relationships was based on α = 0.05.

The Pearson correlations between all GHG and soil properties and crop yield affected by effective variables are presented in Figure 5. The results showed a positive correlation between all gases and GWP with a determined coefficient (R^2^) as follows: N_2_O vs. GWP (0.76), CH_4_ vs. GWP (0.59), and CO_2_ vs. GWP (0.41). Also, there was a negative correlation between GHGI and crop yield with R^2^ = −0.44. The total porosity showed a negative correlation with CH_4_ (R^2^ = −0.23), while positive correlations with other GHG indices were as follows: TP vs. N_2_O (0.27), TP vs. CO_2_ (0.26), TP vs. GWP (0.23), TP vs. CEE (0.21), and TP vs. GHGI (0.26). It should be noted that there was a positive correlation between available nitrate and ammonium vs. crop yield, with an R^2^ of 0.33 and 0.46.

## 3. Discussion

### 3.1. Fertilization System Contribution Effects

According to the literature, any type of fertilization more or less results in gas emission flow, which can be caused by increased plant growth, root extension, and microorganism respiration [21,22]. This is why all types of fertilization systems showed a positive effect size in GHG emission indices (Figure 1). Decomposition of organic matter could represent the main source of methanogenic substrates, which would stimulate the synthesis of CH_4_ [23]. The potential for fertilization to increase nutrient supplies for microbial activity is demonstrated by the higher CH_4_ emissions in all three types of fertilization systems. Some research has shown that applying both organic and conventional fertilization systems has no discernible impact on the CH_4_ emissions from the soil [22,24]. However, contrasting findings regarding CH_4_ emissions have been reported across studies, which can be attributed to differences in the soil moisture, texture, aeration, and microbial community composition. For instance, organic amendments can enhance methanogenic substrate availability, but in well-drained or aerated soils, this may not translate to increased CH_4_ flux due to the enhanced oxidation of methane by methanotrophic bacteria. Conversely, in poorly drained or compacted soils, the same organic inputs can stimulate CH_4_ production [25,26]. Additionally, interactions with other environmental factors, such as temperature, pH, and cropping history, can modulate the extent of methane emission, leading to variability among studies [27,28].

Nonetheless, the use of chemical fertilizers increased gas flux, particularly in terms of N_2_O emissions. The main direct element influencing soil N_2_O emissions is soil nitrogen supply, which stimulates nitrifying microorganisms, resulting in N_2_O emissions [29]. Urea (46% N), anhydrous ammonia (82% N), and ammonium nitrate (34% N) are some common examples of nitrogen supplies that are preferably used in conventional fertilization. It is expected that organic amendments are not comparable with chemical fertilizers in terms of nitrogen availability due to their low nitrogen concentration. However, the system of organic fertilization could notably alter the amount of organic matter in the soil directly as well as indirectly by affecting the carbon cycle and the activities of microorganisms, both of which can have an impact on CO_2_ emissions [30]. It has been reported that the conventional fertilization system alone produced higher CO_2_ emissions than no fertilization, but still lower CO_2_ emissions than the organic fertilization system alone, due to the significant amount of organic carbon in soil treated under the organic fertilization system [31]. Therefore, the higher soil CO_2_ emission flux in the semi-organic fertilization system (Figure 1) could be interpreted by the contribution of adequate soil organic carbon in organic fertilizers (Figure 2). Also, the positive correlation of CO_2_ emissions and soil carbon indices supports this claim (Figure 5). On the other hand, the physical qualities of soil are influenced by soil organic carbon [32]. The observed inverse relationship between soil organic carbon and bulk density (Figure 5) suggests that increased organic carbon enhances soil structure. Lower bulk density corresponds to greater porosity, which plays a crucial role in improving soil aeration [33]. This is why the involvement of organic amendments in fertilization systems results in lower bulk density and higher porosity (Figure 3). It is important to note that these improvements in soil structure typically occur over longer time scales, often requiring multiple cropping seasons or repeated organic amendments to accumulate sufficient organic matter. In contrast, chemical fertilizers can provide more immediate effects on nutrient availability and plant growth, but they generally do not contribute to long-term enhancements in soil physical properties.

In general, organic fertilization systems were shown to have the lowest GWP. Considering so many nutrients fixed in the soil, it has been hypothesized that applying organic nitrogen sources alone has a far lower GWP than using chemical fertilizer alone [34]. Consequently, a lower GHGI in organic fertilization in comparison to conventional and semi-organic systems is inevitable. However, semi-organic systems, despite integrating organic inputs, often exhibit a higher GWP than purely organic systems. This can be attributed to several factors, including the use of synthetic nitrogen fertilizers with high emission potential, the timing and method of fertilizer application that may coincide with periods of high microbial activity, and interactions between readily available inorganic nitrogen and soil organic carbon that can stimulate microbial respiration. Additionally, the combination of fertilizers in semi-organic systems may increase nitrogen availability in early crop growth stages, leading to higher N_2_O emissions and overall GWP compared with organic-only systems. These dynamics highlight the trade-off between enhancing yield and mitigating greenhouse gas emissions in integrated fertilization approaches [35,36,37]. Research has demonstrated that the integration of conventional and organic fertilization approaches can significantly increase crop yields when compared with either system used alone. This suggests that crop growth essential requirements can be supplied by replacing some organic fertilizers with chemical fertilizers [38,39]. With a closer look at the results of crop yield affected by different fertilization systems, it was found that the higher positive effect size of fertilization systems in crop yield was related to the semi-organic fertilization system (Figure 4). In general, organic fertilization is insufficient for crop growth because of its restricted nutrient supply during the early stages of crop growth, and conventional fertilization systems are not good at maintaining soil nutrients [22]. Nevertheless, a combination of applications can continuously increase the nutrient content of the soil while supporting the demands of crop growth [40]. Therefore, an increase in the availability of essential ions for plant growth, such as NH_4_^+^ and NO_3_^−^ as well as base cations, is anticipated. This is the main reason for the higher available soil nutrients in an alkaline environment in semi-organic fertilization (Figure 3). Furthermore, semi-organic fertilization can influence the soil pH dynamics by combining organic inputs that buffer pH fluctuations with inorganic fertilizers that may temporarily acidify or alkalinize the soil, depending on the nitrogen form used (e.g., ammonium vs. nitrate). This buffering effect is particularly relevant in alkaline soils, where maintaining a stable pH can enhance the solubility and availability of key nutrients such as phosphorus, potassium, and micronutrients [41,42]. Additionally, semi-organic systems can modulate nutrient cycling differently across soil types: in clay soils, enhanced cation exchange capacity from organic matter can retain NH_4_^+^ and base cations, whereas in sandy soils, organic inputs improve nutrient retention and reduce leaching [43].

### 3.2. Soil Texture Effects

The interaction of soil mineral particles through aggregation is a key physical process that shapes the chemical, physical, and biological characteristics of soil [44]. Soil texture influences the distribution of aggregate sizes, which in turn affects carbon storage mechanisms and consequently impacts greenhouse gas emissions [45]. As shown in Figure 1, loamy and sandy soils were associated with positive effect sizes for both N_2_O and CO_2_ emissions. This could be due to the soil macro porosity, which is notably high in coarse texture, resulting in higher gas fluxes from soil [46]. It indicates that the pore network in the soil is important for increasing the amount of oxygen available for aerobic bacteria to decompose organic matter, resulting in the production of N_2_O and CO_2_ through microbial respiration [47]. This concept could be additionally supported by the maximum microbial biomass carbon observed in sandy texture (Figure 2). Also, the influence of porosity on microbial activity was confirmed by the positive correlation found between porosity and GHG emission indices (Figure 5). On the other hand, soil with a clay texture produced a lot of CH_4_ emissions. According to reports, clay-textured soils containing a significant number of microaggregates produce greater CH_4_ flux, suggesting that the tightly arranged microaggregates offered suitable anaerobic conditions for the synthesis of methane [48]. The negative correlation between porosity and CH_4_ flux (Figure 5) lends more credence to this since the reduced distribution of macropores may provide a more anaerobic environment. Given that loamy textures increase the potential for GHG emissions, their wide range of pores and particles may be the reason for their maximum GWP and CEE. These features provide the ideal environment for both aerobic and anaerobic bacteria [49,50]. This is why, despite the lack of substantial differences across soil textures in terms of soil carbon indices, loamy and sandy textures produced higher SOC, MBC, CPI, and SOM because of their even distribution of pores, ability to accumulate substrate, and increased activity of microorganisms (Figure 3). Furthermore, increased microbial activity, such as nitrification, was confirmed by the increased availability of nutrients, particularly NH_3_^+^ and NO_3_^−^ ions, which are present in coarse-textured soils and provide substrates for nitrifying bacteria (Figure 4). In terms of crop yields, there was no significant difference between different soil textures. Nonetheless, clay soil performed better in terms of raising crop yield, probably because of its superior capacity to supply steady moisture for plant growth [51]. In contrast to low evaporative demand, where soil dries out gradually and roots have more time to spread in a moist environment, high evaporative demand causes soil to dry out very quickly, which limits crop root growth [52]. The presence of many micropores in fine-textured soils causes water to be held tightly on the surface of silt and clay particles, maintaining a moist rhizosphere for plant roots [44,53]. Consequently, higher crop yields are the result of increased soil nutrient efficiency and plant-necessitated nutrient uptake.

### 3.3. Cropping Strategy Effects

N_2_O emissions highly depend on the abundance of NO_3_^−^ and *Gemmatimonadete* bacteria. *Gemmatimonadete* encompasses denitrifies bacteria capable of converting soil NO_3_^−^ into N_2_ or N_2_O, which causes a depletion of nitrogen and a decrease in crop production [54]. According to reports, the synergistic relationship between this bacterium and root exudates in rhizobium under sole cropping is what causes the abundance of *Gemmatimonadete* in sole cropping vegetables to be noticeably higher than in intercropping systems [55,56]. This is why N_2_O emissions showed a higher effect size in the sole cropping system (Figure 1) and lower available NO_3_^−^ in the soil (Figure 3). Crop rotation and intercropping systems assist in increasing the amount of nitrogen-fixing bacteria in the soil and enhance the plants’ capacity of taking up N from the soil as NO_3_^−^ and NH_4_^+^ [57]. The benefits of intercropping and crop rotation have been extensively shown in a variety of plants such as oat/sunflower and maize/wheat intercropping systems [58,59] and legume-nonlegume crop rotation systems [60,61]. Additionally, the infrastructure and area controlled in sole cropping is twice that of other cropping systems. The greater CO_2_ emissions in sole cropping could be mostly attributed to the use of synthetic fertilizers and the consumption of diesel fuel in tillage processing [62]. In a crop rotation system, the soil’s organic matter availability would significantly increase as a result of the accumulation of root remnants from previously cultivated plants, which have a direct impact on microbial activity [63]. Maintaining plant leftovers increases the amount of organic matter added to the crop rotation strategy, which affects the carbon cycle, and in turn, the gas fluxes [64]. Additionally, crop rotation often involves the incorporation of diverse crop residues that differ in carbon-to-nitrogen ratios, which can stimulate heterotrophic microbial activity and accelerate decomposition, resulting in higher CO_2_ emissions compared with intercropping systems [65,66]. The alternation of crops in rotation also leads to fluctuations in soil microclimate (e.g., moisture and temperature) and microbial community composition, further promoting CO_2_ flux. In contrast, intercropping systems maintain more continuous ground cover and root interactions, which can stabilize soil organic matter and microbial activity, thereby moderating CO_2_ emissions [67,68]. Given that soil fertility is linked to a greater accessibility of nutrients for soil microbial activity and CO_2_ release, it seems logical that there would be a direct relationship between soil microbial biomass carbon and CO_2_ [69]. The explanation for the crop rotation systems’ higher CO_2_ emissions and CEE compared with intercropping systems may be due to this, even though there was no statistically significant difference in the soil C indices between the two types of systems (Figure 1). It has been demonstrated that considering the soil carbon stock, the intercropping system emitted less CO_2_ (690 kg CO_2_-eq ha^−1^) than the sole cropping (1380 kg CO_2_-eq ha^−1^) [62]. The findings of the meta-analysis indicate that there was no significant difference in CH_4_ emissions between cropping strategies. This is most likely because the methanogenic activities that produce the CH_4_ flux are primarily influenced by other variables such as tillage practices and soil water contents [70,71].

Several findings also showed that in comparison to sole cropping, intercropping promotes both productivity and the intake of nutrients [72,73]. As a result, compared with sole cropping, intercropping absorbs considerably more nutrients from the soil. Research indicates that intercropping maize and fava beans can increase phosphorus absorption by up to 29% when compared with sole maize cultivation. The same results were reported for barley/maize, legume/cassava, and maize/fava bean intercropping in terms of nitrogen [74,75]. Additionally, compared with a sole cropping system, crop rotation increased the crop yield and nutrient availability. This can be explained by the advantages of various rhizobium and the benefits of their microorganisms in crop rotation and intercropping. According to previous studies, the plant remnant in crop rotation considerably reduced the number of harmful bacteria and fungi and raised the number of helpful bacteria [76]. However, because of the varied types of root exudates and litter quality, this effect depends on the types of crop and the period of rotation [55]. According to several studies, crop rotation systems outperform sole cropping in terms of crop yield [55,77]. When compared with continuous maize production, it has been observed that the intercropping systems of maize/fava bean, maize/soybean, and maize/chickpea boosted crop output and nutrient intake by approximately 25% [75]. Also, compared with sole cropping, tomato yields improved by 26% in tomato/ryegrass intercropping [78].

### 3.4. Type of Crop Effects

Soil nutrient supplies are important determinants of soil quality and microbial activity. Studies have indicated that vegetable crops—especially leafy varieties such as celery and Chinese cabbage—significantly enhance the soil nutrient levels and stimulate biological activity [55]. This is the main reason for higher nutrient availability and crop yield in vegetables (Figure 3 and Figure 4). Furthermore, several previous investigations have shown that the primary factor controlling soil microbial and enzyme activities is the C and N supplied from roots [79,80]. Leafy vegetable cultivation has been shown to promote the proliferation of beneficial microbial groups, many of which contribute to plant health by suppressing pathogens and enhancing growth [55]. Among these, Proteobacteria play a vital role by acquiring nutrients and combating plant diseases. They are capable of metabolizing a wide array of carbon sources including compounds from root exudates and organic residues [81]. *Actinobacteria* also contribute to disease suppression by synthesizing a broad spectrum of antibiotics that inhibit the development of many plant pathogens [82]. Additionally, higher vegetable yields may be attributed to biochemical defenses in certain crops—such as *Brassicaceae* species—that produce *glucosinolates*. Upon decomposition, these compounds release isothiocyanates, which are known to suppress soilborne pathogens like *Fusarium oxysporum*, thereby supporting improved plant productivity [83]. In contrast, vegetables may also have an impact on the variety of harmful microorganisms present in the soil. Some studies have reported that certain non-leafy vegetable crops—such as cucumber and eggplant—may promote the proliferation of potentially harmful soil bacteria including *Nitrospirae* and *Gemmatimonadetes* [84,85]. These microbial groups are actively involved in the oxidation of ammonia and nitrite, processes that contribute to nitrous oxide (N_2_O) emissions and may also deplete the soil nitrogen levels. This microbial activity could help explain the elevated N_2_O emissions observed in vegetable production systems, as shown in Figure 1.

Although no statistically significant differences in gas emission metrics were detected between cereal and orchard cropping systems, cereals exhibited higher positive effect sizes for CH_4_, CO_2_, global warming potential (GWP), and carbon emission efficiency (CEE). This disparity is likely due to differences in the fertilizer application rates across the two systems [86]. For example, the use of both manure and synthetic nitrogen fertilizers in cropland is a major contributor to soil-derived N_2_O emissions [87]. According to reports, wheat cultivation produced 1.8 times as many N_2_O emissions as the peach orchard [88]. Furthermore, greater N_2_O and CH_4_ emissions are caused by irrigation management, which is far more common on farmland than in orchards, particularly in high-demand crops like rice [89]. In addition, fertilizers are only applied below the seedlings in orchards, which is distinct from spreading fertilizer throughout cereal fields. It should be mentioned that there was a strict limitation on the quantity of orchard data that was accessible and could meet the requirements of our meta-analysis. Therefore, the increased GWP and soil carbon indices in cereals compared with orchards could be justified by these reasons (Figure 1 and Figure 2). When evaluating orchard and cereal systems, greenhouse gas intensity (GHGI)—which accounts for both emissions and crop yield—offers a more meaningful comparison than GWP alone, which can be misleading. Although GWP values did not differ significantly between cereals and orchards, Figure 4 shows that cereal crops had a notably higher yield, with effect sizes of 18.9% for cereals compared with just 1.7% for orchards. This indicates that cereal production leads to a considerably lower carbon intensity per unit of yield compared with orchard cultivation.

## 4. Material and Methods

### 4.1. Literature Survey and Eligibility Criteria

To conduct a structured and comprehensive literature review, data were gathered from three major scientific databases: Web of Science, Scopus, and Crossref. The focus of this study was on exploring the interactions between various fertilization strategies and their effects on greenhouse gas emissions, soil quality, and crop productivity. Accordingly, a set of targeted keywords was employed to identify relevant publications. These included terms such as organic farming, organic fertilization, chemical fertilization, conventional agriculture, semi-organic fertilization, fertilizer practices, greenhouse gas emissions, soil health, crop yield, soil nutrients, plant productivity, crop types, combined fertilization, cropping systems, agricultural strategies, soil texture, monoculture, sole cropping, crop rotation, intercropping, mixed cropping, soil carbon indices, and nutrient management.

To ensure comparability and rigor in the meta-analysis, the following selection criteria were applied: (1) studies must report both control (no fertilization) and treatment (with fertilization) conditions; (2) studies lacking replication in control and treatment groups were excluded; (3) only articles published in peer-reviewed journals were considered; (4) publications had to be in English; (5) data had to be available for at least two of the key variables of interest.

The majority of the data were obtained directly from tables presented in the selected studies. For information displayed in graphical form, values were extracted using the software GetData Graph Digitizer (version 2.24).

### 4.2. Collection of Data

**Figure 6:**
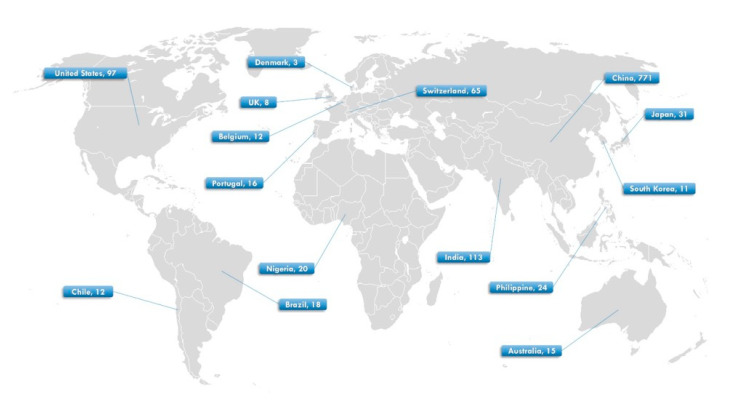
Global distribution map showing the locations of the paired observations included in the meta-analysis.

Out of approximately 700 papers initially screened, 58 met all the inclusion criteria and were selected for analysis. From these studies, a total of 1216 paired observations (effect sizes) were extracted. The worldwide distribution of observations is illustrated in Figure 6.

To investigate how various fertilization approaches influence greenhouse gas emissions, crop yield, and soil health, the data were organized into two main categories: (1) influencing factors and (2) response variables.

In the first data category, we identified four key factors to guide the literature review: (i) fertilization system, (ii) soil texture, (iii) cropping system, and (iv) plant type. Fertilization systems were grouped into three categories: (1) organic (e.g., manure, compost, bio-organic amendments), (2) conventional (e.g., chemical fertilizers such as urea), and (3) semi-organic, which involves a combination of organic and chemical fertilizers (e.g., NPK plus manure or compost plus urea). Soil texture was classified into three groups: (1) clayey soils (including sandy clay, silty clay, etc.), (2) loamy soils (such as clay loam, silt loam), and (3) sandy soils (e.g., loamy sand, sandy loam). The cropping system was divided into (1) crop rotation, (2) sole cropping, and (3) intercropping. Finally, plant types were categorized as (1) cereals (e.g., rice, wheat), (2) vegetables (e.g., cabbage, lettuce), and (3) orchards (e.g., citrus, apple).

The second data category comprised the affected variables, which were grouped into four classes: (i) greenhouse gas emission indicators including N_2_O, CH_4_, CO_2_, global warming potential (GWP), carbon emission efficiency (CEE), and greenhouse gas intensity (GHGI); (ii) soil carbon indicators such as soil organic carbon (SOC), microbial biomass carbon (MBC), and carbon pool index (CPI); (iii) soil properties including bulk density (BD), total pore volume (TPV), organic matter (OM), ammonium (NH_4_^+^), nitrate (NO_3_^−^), total nitrogen (TN), available phosphorus (Av. P), available potassium (Av. K), and pH; and (iv) crop yield.

To facilitate comparisons across studies, all extracted data were standardized to consistent units: GHG emissions in kg ha^−1^ year^−1^; SOC in g kg^−1^; MBC in mg kg^−1^; CPI in %; BD in mg kg^−1^; TP in %; OM in g kg^−1^; NH_4_^+^ and NO_3_^−^ in µg g^−1^; TN in g kg^−1^; available P and K in mg kg^−1^; pH measured in H_2_O; and crop yield in kg ha^−1^. The indices GWP and GHGI were calculated based on the measured N_2_O, CH_4_, CO_2_ emissions, and crop yield data. Global warming potential (GWP) quantifies the relative contribution of greenhouse gases to atmospheric warming [90]. Specifically, methane (CH_4_) and nitrous oxide (N_2_O) have global warming potentials approximately 25 and 298 times greater than carbon dioxide (CO_2_), respectively [91]. These values are expressed as CO_2_-equivalent emissions, with GWP calculated using the following formula [90]:

$$GWP(kg\;{CO}_{2}\;{ha}^{-1})={CO}_{2}flux+{(N}_{2}O\;flux\times 298)+({CH}_{4}flux\;\times 25)$$

Also, GHGI, which represents the overall carbon intensity per ton of crop yield (t CO_2_ eq t^−1^), was calculated as follows: GHGI = GWP/crop yield.

### 4.3. Meta-Analyses

A meta-analysis quantifies the degree of change in a variable and assesses its significance in relation to another variable. This change is measured as the effect size. In this study, the effect size was calculated using the natural logarithm of the response ratio (RR), following the method described by [92]:

$$ln(RR)=ln(\frac{X_{T}}{X_{C}})$$

In this context, *X_C_* and *X_T_* represent the mean values of a variable for the control and treatment groups, respectively. The response ratio (*RR*) can be interpreted as (e^ln(*RR*)^ − 1) × 100, which expresses the percentage change attributable to the influencing factors [93]. For each parameter—greenhouse gases, carbon indices, soil properties, and crop yield—we collected the standard deviation (SD) and sample size (n) from both the control and treatment groups to calculate confidence intervals (CIs) around the effect sizes. When only the standard error (SE) or coefficient of variation (CV) was reported, the SD was calculated using the formulas: SD = SE × √n and SD = CV × mean. The statistical significance of effect sizes was evaluated using 95% confidence intervals; a difference between groups was considered significant if the 95% CI did not include zero (*p* ≤ 0.05).

### 4.4. Data Analyses

Effect sizes and 95% confidence intervals for each categorical group were calculated using SPSS version 28 (IBM Statistics, New York, NY, USA). Based on the heterogeneity test results, a random effects model was applied. Groups with fewer than three pairwise comparisons were excluded from the analysis. Resampling procedures were performed with 999 iterations to enhance robustness. To assess potential publication bias and the overall reliability of the meta-analysis, funnel plot statistics and Rosenthal’s Fail-safe N method were employed. The Fail-safe N values were compared against the threshold 5n + 1 (where n is the number of cases) only when the funnel plot statistics (Kendall’s Tau and Spearman Rank-Order correlation) indicated significance (*p* < 0.05). No evidence of publication bias was detected in the dataset. Between-group heterogeneity was evaluated using the Qb statistic and corresponding *p*-values within the random-effects framework to examine the influence of fertilization methods on GHG emissions, carbon metrics, soil characteristics, and crop yields. Pearson correlation tests were conducted to analyze relationships among the affected variables. Autocorrelation was checked using the Durbin–Watson test. Model residuals passed the Cook–Weisberg test, confirming no heteroscedasticity or influential outliers. The normality of residuals was verified by the Shapiro–Wilk test (*p* < 0.05). Additionally, the overall regression model significance was supported by the Fisher–Snedecor F-test, with residuals showing an approximate normal distribution and mean values near zero.

## 5. Conclusions

The findings of this meta-analysis suggest that semi-organic fertilization, which combines organic and chemical inputs, can outperform conventional systems in crop productivity while maintaining comparable GHG intensity. This approach may offer a cost-effective alternative by reducing the dependence on synthetic fertilizers and promoting long-term soil nutrient balance, ultimately contributing to lower emissions. Integrating organic amendments as partial substitutes for chemical fertilizers appears to enhance nutrient use efficiency and reduce carbon intensity. Additionally, diversified cropping systems like rotation and intercropping, by recycling plant residues, can improve yields, reduce GHG outputs, and decrease fertilizer-related costs. While these insights are particularly valuable for optimizing cereal production systems, the analysis also underscores the promise of vegetable cultivation in reducing the GHG emissions per unit of output, offering a pathway toward more sustainable agricultural practices.

## Data Availability

The original contributions presented in this study are included in the article. Further inquiries can be directed to the corresponding author.
